# ESKtides: a comprehensive database and mining method for ESKAPE phage-derived antimicrobial peptides

**DOI:** 10.1093/database/baae022

**Published:** 2024-03-26

**Authors:** Hongfang Wu, Rongxian Chen, Xuejian Li, Yue Zhang, Jianwei Zhang, Yanbo Yang, Jun Wan, Yang Zhou, Huanchun Chen, Jinquan Li, Runze Li, Geng Zou

**Affiliations:** National Key Laboratory of Agricultural Microbiology, College of Biomedicine and Health, Huazhong Agricultural University, Shenzhen Institute of Nutrition and Health, Huazhong Agricultural University, Shizishan Street No. 1, Wuhan 430070, China; Hubei Hongshan Laboratory, College of Food Science and Technology, Huazhong Agricultural University, Shizishan Street No. 1, Wuhan 430070, China; National Key Laboratory of Agricultural Microbiology, College of Biomedicine and Health, Huazhong Agricultural University, Shenzhen Institute of Nutrition and Health, Huazhong Agricultural University, Shizishan Street No. 1, Wuhan 430070, China; Hubei Hongshan Laboratory, College of Food Science and Technology, Huazhong Agricultural University, Shizishan Street No. 1, Wuhan 430070, China; National Key Laboratory of Agricultural Microbiology, College of Biomedicine and Health, Huazhong Agricultural University, Shenzhen Institute of Nutrition and Health, Huazhong Agricultural University, Shizishan Street No. 1, Wuhan 430070, China; Hubei Hongshan Laboratory, College of Food Science and Technology, Huazhong Agricultural University, Shizishan Street No. 1, Wuhan 430070, China; College of Informatics, Huazhong Agricultural University, Shizishan Street No. 1, Wuhan 430070, China; National Key Laboratory of Agricultural Microbiology, College of Biomedicine and Health, Huazhong Agricultural University, Shenzhen Institute of Nutrition and Health, Huazhong Agricultural University, Shizishan Street No. 1, Wuhan 430070, China; Hubei Hongshan Laboratory, College of Food Science and Technology, Huazhong Agricultural University, Shizishan Street No. 1, Wuhan 430070, China; National Key Laboratory of Crop Genetic Improvement, Shizishan Street No. 1, Wuhan 430070, China; College of Informatics, Huazhong Agricultural University, Shizishan Street No. 1, Wuhan 430070, China; Hubei Hongshan Laboratory, College of Food Science and Technology, Huazhong Agricultural University, Shizishan Street No. 1, Wuhan 430070, China; College of Fisheries, Huazhong Agricultural University, Shizishan Street No. 1, Wuhan 430070, China; National Key Laboratory of Agricultural Microbiology, College of Biomedicine and Health, Huazhong Agricultural University, Shenzhen Institute of Nutrition and Health, Huazhong Agricultural University, Shizishan Street No. 1, Wuhan 430070, China; College of Veterinary Medicine, Huazhong Agricultural University, Shizishan Street No. 1, Wuhan 430070, China; National Key Laboratory of Agricultural Microbiology, College of Biomedicine and Health, Huazhong Agricultural University, Shenzhen Institute of Nutrition and Health, Huazhong Agricultural University, Shizishan Street No. 1, Wuhan 430070, China; Hubei Hongshan Laboratory, College of Food Science and Technology, Huazhong Agricultural University, Shizishan Street No. 1, Wuhan 430070, China; College of Veterinary Medicine, Huazhong Agricultural University, Shizishan Street No. 1, Wuhan 430070, China; Shenzhen Branch, Guangdong Laboratory for Lingnan Modern Agriculture, Genome Analysis Laboratory of the Ministry of Agriculture and Rural Affairs, Agricultural Genomics Institute at Shenzhen, Chinese Academy of Agricultural Sciences, Buxin Road No. 97, Shenzhen 518000, China; Shenzhen Institute of Quality & Safety Inspection and Research, Buxin Road No. 97, Shenzhen 518000, China; National Key Laboratory of Agricultural Microbiology, College of Biomedicine and Health, Huazhong Agricultural University, Shenzhen Institute of Nutrition and Health, Huazhong Agricultural University, Shizishan Street No. 1, Wuhan 430070, China; Hubei Hongshan Laboratory, College of Food Science and Technology, Huazhong Agricultural University, Shizishan Street No. 1, Wuhan 430070, China; National Key Laboratory of Agricultural Microbiology, College of Biomedicine and Health, Huazhong Agricultural University, Shenzhen Institute of Nutrition and Health, Huazhong Agricultural University, Shizishan Street No. 1, Wuhan 430070, China; Hubei Hongshan Laboratory, College of Food Science and Technology, Huazhong Agricultural University, Shizishan Street No. 1, Wuhan 430070, China

## Abstract

**‘Superbugs’ have received increasing attention from researchers, such as ESKAPE bacteria (*Enterococcus faecium, Staphylococcus aureus, Klebsiella pneumoniae, Acinetobacter baumannii, Pseudomonas aeruginosa* and *Enterobacter* spp.), which directly led to about 1 270 000 death cases in 2019. Recently, phage peptidoglycan hydrolases (PGHs)–derived antimicrobial peptides were proposed as new antibacterial agents against multidrug-resistant bacteria. However, there is still a lack of methods for mining antimicrobial peptides based on phages or phage PGHs. Here, by using a collection of 6809 genomes of ESKAPE isolates and corresponding phages in public databases, based on a unified annotation process of all the genomes, PGHs were systematically identified, from which peptides were mined. As a result, a total of 12 067 248 peptides with high antibacterial activities were respectively determined. A user-friendly tool was developed to predict the phage PGHs–derived antimicrobial peptides from customized genomes, which also allows the calculation of peptide phylogeny, physicochemical properties, and secondary structure. Finally, a user-friendly and intuitive database, ESKtides (** http://www.phageonehealth.cn:9000/ESKtides), **was designed for data browsing, searching and downloading, which provides a rich peptide library based on ESKAPE prophages and phages**.

**Database URL:**  10.1093/database/baae022

## Introduction

Over the past decades, bacterial antimicrobial resistance has emerged as a significant global threat to human health. It was estimated that by 2050, antimicrobial resistance will cause 10 million deaths each year and an estimated direct economic loss of three trillion pounds (1). As early as 2017, WHO identified a list of priority multidrug-resistant pathogens, highlighting multidrug-resistant ESKAPE bacteria (*Enterococcus faecium, Staphylococcus aureus, Klebsiella pneumoniae, Acinetobacter baumannii, Pseudomonas aeruginosa* and *Enterobacter* spp.) as a serious worldwide medical concern (2). In 2019, global ESKAPE infection caused about 929 000 death cases (3). Researchers have proposed the spectrum of drugs faced with the resistance by ESKAPE pathogens, including oxazolidinones, lipopeptides, macrolides, fluoroquinolones, tetracyclines, beta-lactams and beta-lactam-beta-lactamase inhibitor combinations, which cover >90% of the types of antibiotics (4). With traditional antibiotic therapies becoming increasingly ineffective, there is an urgent need for alternative therapeutic agents to combat resistant pathogens.

Antimicrobial peptides (AMPs) have been reported to be effective for microorganisms with resistance to conventional antibiotics (5). Currently, the antibacterial mechanisms of AMPs are divided into two aspects: cell membrane–targeting mechanism that uses hydrophobic action to destroy cell membranes and forms holes to cause cell death (6) and intracellular-targeting mechanisms that destroy cells by interfering with their normal metabolism (7–9). According to previous studies, most natural AMPs are derived from animals, plants, bacteria, fungi, and protozoa, and AMPs derived from virus are rarely reported.

As a kind of virus, phages are abundant and widely distributed in the environment (there are 10^31^ phages in nature). As bacterial hunters, phages can specifically lyse bacteria via phage peptidoglycan hydrolases (PGHs) with strong antibacterial activity, providing novel strategies against multidrug-resistant bacteria (10–12). Recently, Thandar *et al*. analyzed the secondary structure of phage lysin PlyF307, a kind of PGHs, and confirmed the *in vitro* antibacterial activity of the C-terminal peptide P307, which possesses a dual alpha-helical structure (13). This finding suggests the potential for AMP discovery through PGHs. Furthermore, two independent studies have demonstrated that deep learning model can be used to predict the activity of AMPs (14, 15) offering the opportunity to expand the existing AMP dataset by mining phage-derived AMPs based on PGHs and assessing their activity using deep learning models. With advancements in high-throughput culturomics and genome sequencing technologies, an increasing number of phage genomes are being reported. However, comprehensive genome-wide mining techniques for phage or prophage AMPs and dedicated databases for such AMPs are currently lacking.

In this study, we used ESKAPE genome and corresponding phage genome data were used to develop a new computational pipeline for systematic mining of AMPs based on the PGHs detected from ESKAPE isolates and their phages (for ESKAPE phage-derived peptides, https://github.com/hzaurzli/phatides_prediction). The antimicrobial activity, the physiochemical properties and secondary structure of ESKAPE phage-derived AMPs were also evaluated in our database. To facilitate the accessibility and analysis of ESKAPE phage-derived AMPs, a ESKtides database was constructed, which can be accessed at http://www.phageonehealth.cn:9000/ESKtides. The database comprises AMPs mined based on PGHs, which are generally absent in the existing databases related to peptides.

## Materials and methods

**Figure 1.**
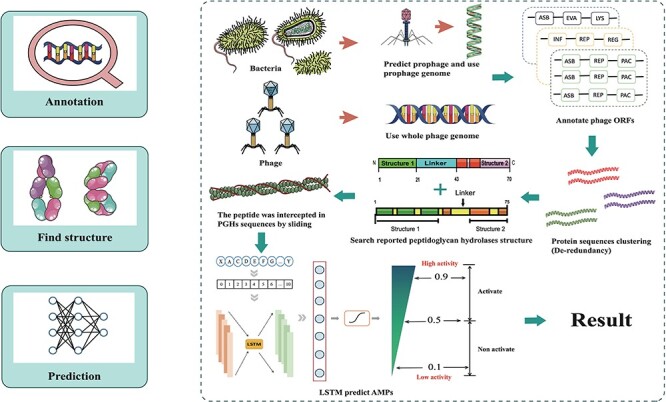
Workflow for ESKAPE-derived peptides mining. The main steps include annotation of phages and prophages, de-redundancy, search PGH structure and scoring of antibacterial activity.

The database was established through data integration, phage and prophage genome annotation, PGHs identification and peptides mining (Figure 1).

### Data collection for the genomes of ESKAPE isolates and their phages

**Figure 2.**
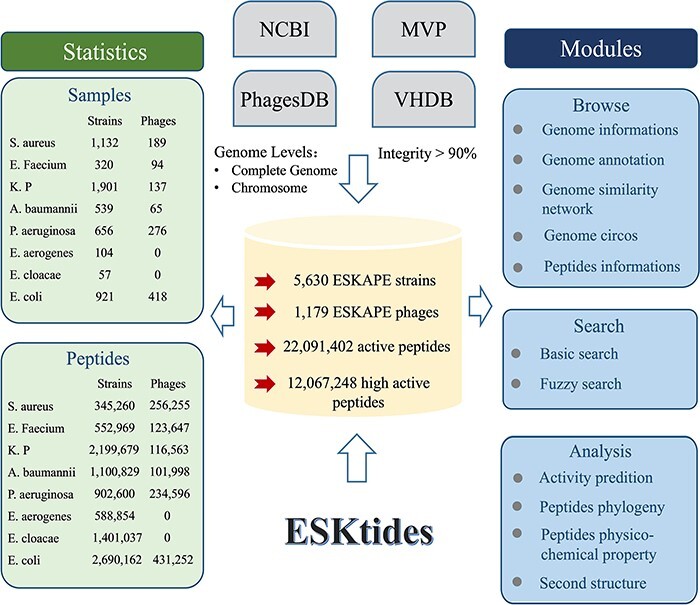
Overall design of ESKtides. ESKtides curated metadata information from NCBI, MVP, VHDB and PhagesDB, and the data in these four databases are subjected to the following procedures. First, for ESKAPE strains, we filter incomplete assembly to ensure the accuracy of mining. Second, for ESKAPE phages, we downloaded the phage genome corresponding to their strains (phages are isolated from corresponding strains) from MVP, PhagesDB, VHDB and marked as corresponding phages. All raw data were processed by using a standard pipeline. ESKtides includes ‘Browse’, ‘Search’, ‘Download’ and ‘Submission’.

The genome data were downloaded (including ESKAPE strains and their phages) from four widely used databases on January 2023, including Microbe Versus Phage (MVP) (16), PhagesDB (17), Virus-Host Database (VHDB) (18) and National Center for Biotechnology Information (NCBI) (19) and merged these genomes to construct our dataset in this study. The data in these four databases were subjected to the following processing. First, genome analysis of ESKAPE strains was conducted to isolate prophage elements, concurrently with the elimination of any incomplete genomic assemblies from the NCBI database, including contigs and scaffolds, to refine the prophage extraction accuracy. Second, for the ESKAPE phage genomes, the genomes of the phages with ESKAPE strains as hosts were collected from MVP, PhagesDB, and VHDB, while the phages from metagenomic data were omitted. Third, CheckM (20) and CheckV (21) were used to evaluate genome integrity and filter the data in which integrity is <90% in order to avoid artificial differences resulting from different annotation pipelines. The genome annotation circos was generated by CGView.js (Figure 2).

### Obtaining peptides from ESKAPE phages and prophages

The initial step in the extraction of ESKAPE phage-derived AMPs is to obtain the peptides from the predicted PGHs within ESKAPE phage and prophage genome sequence. For prophage, each corresponding strain genome open reading frame (ORFs) were done by using Prokka 1.14.6 (22). Phispy 4.2.21 (23) was used to discover prophages based on annotations, and then prophages coordinate was extracted by custom scripts. To reannotated prophage genome, PHANOTATE (24) was performed to annotate prophage ORFs and protein sequences were obtained from ORFs. CD-hit (25) was used to eliminate redundancy and remove proteins in which molecular weight is >40 kDa. Additionally, proteins with previously reported PGH domains were identified through mapping against a database of PGHs compiled from published literature using ‘hmmsearch’ (26). Next, transmembrane proteins are removed which is predicted by DeepTMHMM (27). Finally, we obtain all putative PGHs. To derive peptides from PGHs, sequences ranging in length from 6 to 50 amino acids (aa) were extracted from PGHs using a sliding window cutting method. For phages, Prodigal (28) was performed to annotate phage ORFs, protein sequences were obtained from ORFs directly and the remaining steps are same as strains.

### Activity evaluation and secondary structure prediction

A deep learning model was used to assess the activity of the peptides obtained from PGHs. Under the deep learning frame, the basic model was structured with convolutional neural networks and long short-term memory layers, which has been used in the research of Ma *et al*. (15). The model was established as follows: (i) a non-AMP dataset was collected from UniProt (http://www.uniprot.org), while an AMP dataset was collected from four publicly available sources (Database of Anti-Microbial peptides, Antimicrobial Peptide Database, APD; Collection of Anti-Microbial Peptides, and A Database Linking Antimicrobial Peptides). The data were divided into training and testing sets at an 8:2 ratio. (ii) Key layers and parameters were configured, including an embedding layer with an input dimension of 21, an input length of 300 and an output dimension of 128; a 1D convolution layer with 64 convolution kernels, 16 filter length and ReLU activation function; a 1D max pooling layer with a pool size of 5; a long short-term memory layer with 100 units; and a dense layer with one unit and sigmoid activation function. (iii) The training set was used to train the model built with the Keras framework (version 2.2.4, https://www.keras.io). Secondary structure predictions were calculated using SCRATCH-1D 1.1 (29).

### Evaluate the physiochemical properties of the peptide

We also provide a platform for users to calculate physicochemical property by using R package ‘Peptides’ (https://www.rdocumentation.org/packages/Peptides/versions/2.4.4) and Biopython (version 1.79). Protein length, molecular weight, instability, hydrophobicity, hydrophobic moment, aliphatic, pI and charge were included in our platform by using different functions: ‘lengthpep()’, ‘mw()’, ‘instaIndex()’, ‘hydrophobicity()’, ‘hmoment()’, ‘aIndex()’, ‘pI()’ and ‘charge()’ in R package Peptides. Protein gravy is calculated by function ‘gravy()’ in Biopython.

## Results

### Data summary

Several peptide-related databases have been reported in recent studies, and their differences from ESKtides are summarized in Table 1. APD3 is an AMP database sourced from archaea, protists, fungi, plants and animals (30). 3.0 Data Repository of Antimicrobial Peptides (DRAMP) provides information of AMPs from clinical, general and patent sources as well as AMPs mined from bacteriocins and plants (31). DBAASP (32) and R1–R4 Collection of Anti-Microbial Peptides (CAMPR) (33–36) comprise manually collected and experimentally verified high-quality peptides, resulting in a more limited dataset compared to those derived from high-throughput methods. Notably, ESKtides stands as the inaugural extensive phage-derived AMP database.

**Table 1.** Comparison between ESKtides and relevant databases

Database	Data source	Activity score	Physical and chemical properties	Sequence structure	Mining by users	Peptides design
ESKtides	Phages and prophages	Yes	Yes	Yes	Yes	No
APD	Archaea, protists, fungi, plants and animals	No	No	No	No	Yes
APD 2.0	Archaea, protists, fungi, plants and animals	No	No	No	No	Yes
APD 3.0	Archaea, protists, fungi, plants and animals	No	Yes	No	No	Yes
DRAMP	Bacteriocins, clinical, patent, plant and stapled AMPs	Yes	No	No	No	No
DRAMP 2.0	Bacteriocins, clinical, patent, plant and stapled AMPs	Yes	No	No	No	No
DRAMP 3.0	Bacteriocins, clinical, patent, plant and stapled AMPs	Yes	No	No	No	No
DBAASP	Not clear	No	Yes	No	No	No
CAMPR R1–R4	Not clear	Yes	No	No	No	No

### Pipeline and ESKAPE phage-derived peptides of ESKtides

The mining approach of ESKtides has been described in the Methods and Material section, with the technical details and schematic shown in Figure 1. In general, the process comprises three key stages: (1) annotating of phage and prophage genomes; (2) identifying PGHs based on their possession of protein functional domains from previously reported PGHs, followed by the extraction of peptides from the predicted PGHs; (3) evaluating the antibacterial activity of the peptides using a deep learning method.

ESKtides comprises a total of 12 067 248 AMPs, selected based on the predicted antibacterial activity (the cutoff value = 0.9) from a pool of 22 091 402 peptides abstracted from 185 177 PGHs identified in the prophages of 5630 ESKAPE isolates and 1179 ESKAPE phages. The efficiency of AMP mining (measured as the number of AMPs per genome) varies significantly between different species. For ESKAPE isolates, *E. cloacae* exhibits the highest average number of AMPs per genome (24 579 peptides per genome), while *S. aureus* presents the lowest (305 peptides per genome). For ESKAPE phages, *E. faecium* phages have the maximum efficiency (1315 peptides per genome) and *P. aeruginosa* phages have the minimum efficiency (850 peptides per genome).

### Database access

**Figure 3.**
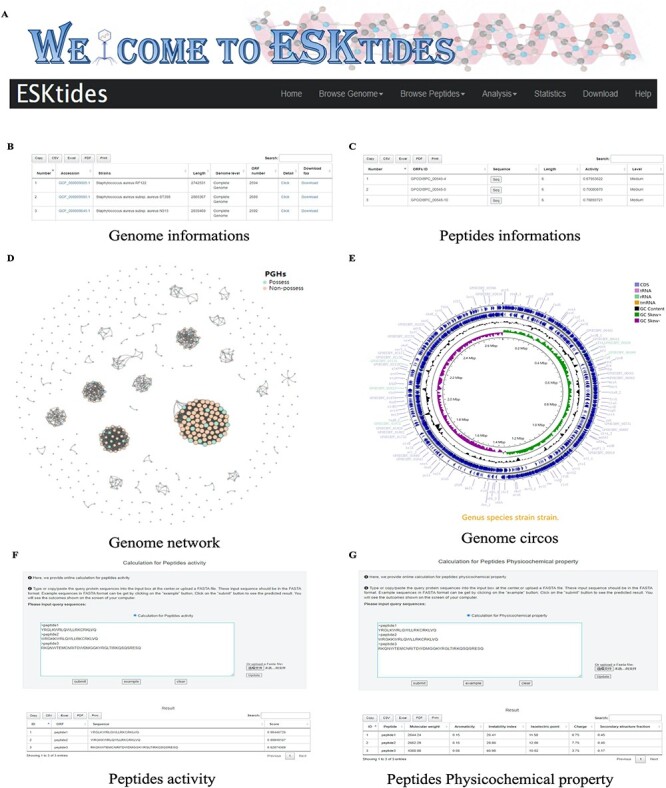
Overview of ESKtides. (A) Main functions of ESKtides, including the ‘Browse Genome’, ‘Browse Peptides’, ‘Analysis’, ‘Statistic’, ‘Download’ and ‘Help’ modules. (B) A table of queried strains or phages information in the ‘Browse Genome’ module. (C) A table of queried ESKAPE-derived peptides information in the ‘Browse Peptides’ module. (D) A graph of queried PGH distribution in the ‘Browse Genome’ module. (E) The annotation circos graph of queried strain. (F) Submit peptides sequences to score the bactericidal activity of the peptide. (G) Calculate peptides sequences physicochemical property.

A user-friendly website was designed for the database, with five main modules, including ‘Browse Genome’, ‘Browse Peptides’, ‘Analysis’, ‘Statistic’ and ‘Download’ (Figure 3A). In each table, search boxes are designed for users to perform quick search with two patterns: exact search and fuzzy search. ESKAPE phage-derived peptide pipeline is shown on the home page for users to better understand the whole mining process.

On the page of ‘Browse Genome’, users can browse ESKAPE profiles of different strains and phages on the top box, search for genome information by accession, strain, genome length, genome level and ORF number on the center table, and browse genome similarity network on the bottom graph, where each point represents one genome. For example, if users attempt to query genome annotation, they only need to click ‘detail’ button to access the annotation page, which is on the center table. The searching results will be displayed in a table containing ‘Locus tag’, sequence type, ‘CDS length’, ‘gene name’, ‘EC number’, ‘COG id’ and ‘product function’ (Figure 3B). In addition, users can browse genome annotation circos plot on the top and can also download the annotated proteins fasta files by clicking ‘Download’ (Supplementary Figure.S4).

On the page of ‘Browse Peptides’, users can view the ESKAPE phage-derived peptides from different ESKAPE strains or phages and can also search for peptide information by ‘ORFs ID’, ‘Sequence’, ‘peptides length’, ‘peptides activity’ and ‘activity level’. The antibacterial activity value is within the range of 0–1. Two levels are set for activities: if the peptide activity is higher than 0.9, it is defined as ‘High’; if the peptide activity is between 0.5 and 0.9, it is defined as ‘Medium’; otherwise, the peptides are not included in our dataset. Users can select ESKAPE phage-derived peptides of interest and click the ‘Seq’ button to download the information (Figure 3C). Group information includes ‘ORFs ID’, ‘Sequence’, peptide length, peptide activity and activity level. Users can also download the information of all ESKAPE phage-derived peptides on the ‘Download’ page in corresponding group datasets that users are interested in.

On the page of ‘Analysis’, in ‘Peptides activity Prediction’, users can predict the antibacterial activity of peptides by using the deep learning model. Users can start a new calculation after clicking the ‘submit’ button and click the ‘example’ button to show the example format. The ‘clear’ button can be used to erase the previous record. In ‘Peptides Phylogenetic tree’, users can show the phylogenetic relationship of related peptides. Users only need to upload a tree file (newick format or json format) and adopt different styles according to their needs. We also provide a calculation platform for the physicochemical properties of peptides, which is used in the same way as ‘Peptides activity Prediction’. The secondary structure can also be predicted in ‘Peptides secondary structure’ by using SCRATCH-1D_1.1. All operations can be performed in batches (Supplementary Figure S5).

All analysis results can be downloaded as comma-separated values (CSV) files for each group on the ‘Download’ page, and all search and calculation results can be downloaded as CSV and Excel files for customized analysis by clicking the corresponding button on the top right of almost all tables.

Users can submit relevant data by sending us a data information table via email. Currently, ESKtides can accept open access genome fasta files and assembly fasta files related to ESKAPE. The submitted data will be added to ESKtides after curation and analysis as described in Materials and Methods section. We also provide the mining pipeline for ESKAPE phage-derived peptides. The software is available on GitHub: https://github.com/hzaurzli/phatides_prediction.

### Usage example

A case study was conducted to estimate the performance of ESKtides and then predicted the reported peptides based on PGHs. The AMP sequences recorded in relevant papers that confirmed by biological experiments before 1 January 2023 were collected. Then, the peptide activity was predicted by ESKtides and compared the prediction score with the results of biological experiments (Table 2). These predicted peptide activities, sorted by prediction scores, are listed in Table 2. Comparative analysis with CAMPR4 showcased advantages in our platform, particularly in PGH-based peptide performance prediction. For example, the studies (37, 38) described that *P. aeruginosa* peptides (X1–X4 and PaP1-1 from PGHs) show low antibacterial activity in experiment, but CAMPR4 was determined to be high activity, our platform was determined the low activity, which is more fitted with experiment result. This case study underscores our platform’s efficacy in peptide identification and streamlining candidates for subsequent biological experiments.

**Table 2.** Usage example, analysis of 10 peptides, which are experimentally verified

Source	Peptides	Experimental bactericidal activity	Activity score (ESKtides)	Activity score (CAMPR4)	Reference
*P. aeruginosa* PGHs (D3 ORF31)	X1	Low	0.029	0.81 (RF) 0.69 (SVM)	(38)
*P. aeruginosa* PGHs (D3 ORF31)	X2	Low	0.016	0.85 (RF) 0.85 (SVM)	(38)
*P. aeruginosa* PGHs (D3 ORF31)	X3	Low	0.016	0.84(RF) 0.82(SVM)	(38)
*P. aeruginosa* PGHs (D3 ORF31)	X4	Low	0.26	0.53 (RF) 0.30 (SVM)	(38)
*P. aeruginosa* PGHs (PlyPa01)	PaP1-1	Low	0.382	0.64 (RF) 0.50 (SVM)	(37)
*P. aeruginosa* PGHs (PlyPa01)	PaP1-2	Low	0.757	0.82 (RF) 0.62 (SVM)	(37)
*A. baumannii* PGHs (PlyF307)	P307	Good	0.999	0.94 (RF) 0.96 (SVM)	(39)
*A. baumannii* PGHs (LysP53)	P103	Low	0.005	0.29 (RF) 0.07 (SVM)	(13)
*A. baumannii* PGHs (LysP53)	P104	Good	0.029	0.06 (RF) 0.01 (SVM)	(13)
*Mycobacterium* PGHs	AK15	Excellent	0.937	0.97 (RF) 1.00 (SVM)	(40)

Abbreviations: RF: Random Forests; SVM: Support Vector Machines.

## Discussion

In recent years, several peptide-related databases such as DRAMP 2.0 (39), DRAMP 3.0 (31), APD3 (30) and CAMPR R1–R4 (33–36) have been established. However, there has been limited research on mining AMPs from phages. In this study, ESKtides was established, offering comprehensive insights into AMPs derived from ESKAPE prophages and phages. To the best of our knowledge, ESKtides is the first comprehensive database of ESKAPE phage-derived AMPs, which may expand the knowledge of AMP source and PGH distribution in ESKAPE isolates and phages. Remarkably, the database provides useful tools such as ‘Peptides Activity Prediction’ and ‘Peptides Phylogenetic Tree’, alongside the computation of physicochemical properties, to assist users in selecting more suitable peptides. In this version of ESKtides, a deep learning model was utilized to predict the antibacterial activity of peptides. In comparison to CAMPR4, ESKtides, based on the deep learning model in Ma *et al*. (15), has more advantages in predicting peptides mined based on PGHs. Previous studies (37, 38) described that *P. aeruginosa* peptides (X1–X4 and PaP1-1 from PGHs) showed low antibacterial activity in experiments, in contrast to the high activity predicted by CAMPR4. ESKtides showed more closely with experimental results by indicating low activity for these peptides. These results demonstrate that ESKtides performs well in identifying peptides and narrowing the scope of candidates for further biological experiments. However, because only using the deep learning model to predict the peptide activity is likely to introduce some false positives, the database probably incorporates some ESKAPE phage-derived peptides that are less active in the real situation. Therefore, we intend to conduct further validation experiments to reduce the false positive rate. ESKtides promises to be a valuable resource for advancing the understanding of AMPs.

### Database architecture

The ESKtides website runs on an Apache Web server (https://apache.org/). The database was developed by using sqlite3 (https://www.sqlite.org/index.html). Flask 2.0.3 (https://flask.net.cn/) was used for server-side scripting. The ESKtides web interface was built by using Datatables (https://datatables.net/) and JQuery v2.1.1 (http://jquery.com). ECharts (http://echarts.baidu.com) was used as a graphical visualization framework and R (https://www.r-project.org/) for graph drawing. We recommend using the latest versions of Firefox and Google Chrome for the best experience.

## Supplementary Material

baae022_Supp

## Data Availability

ESKtides is freely available to the public without registration or login requirements (http://www.phageonehealth.cn:9000/ESKtides).
