# Correction to: ESKtides: a comprehensive database and mining method for ESKAPE phage-derived antimicrobial peptides

**DOI:** 10.1093/database/baae035

**Published:** 2024-05-06

This is a correction to: Hongfang Wu, Rongxian Chen, Xuejian Li, Yue Zhang, Jianwei Zhang, Yanbo Yang, Jun Wan, Yang Zhou, Huanchun Chen, Jinquan Li, Runze Li, Geng Zou, ESKtides: a comprehensive database and mining method for ESKAPE phage-derived antimicrobial peptides, *Database*, Volume 2024, 2024, baae022, https://doi.org/10.1093/database/baae022.

In the originally published author list for this manuscript, the ‡ symbol (denoting an equal contribution) was erroneously applied to author Runze Li. This error has been corrected.

