# Supplementary material for: ESKtides: a comprehensive database and mining method for ESKAPE phage-derived antimicrobial peptides
Source: Database (Oxford). 2024 Mar 26;2024:baae022. doi: 10.1093/database/baae022 (PMC10965241; doi:10.1093/database/baae022)
Supplement: baae022_Supp [file baae022_supp.zip › suppl_data/Supplementary material 20240226.docx]

**Table S1. ESKAPE dataset**

| **Item** | **Strains** | **Phages** |
| --- | --- | --- |
| *Staphylococcus aureus* | 1,132 | 189 |
| *Enterococcus Faecium* | 320 | 94 |
| *Klebsiella pneumoniae* | 1,901 | 137 |
| *Acinetobacter baumannii* | 539 | 65 |
| *Pseudomonas aeruginosa* | 656 | 276 |
| *Enterobacter aerogenes* | 104 | 0 |
| *Enterobacter cloacae* | 57 | 0 |
| *Escherichia coli* | 921 | 418 |

**Table S2. ESKAPE-derived peptides**

| **Item** | **Strains derived peptides** | **Phages derived peptides** |
| --- | --- | --- |
| *Staphylococcus aureus* | 345,260 | 256,255 |
| *Enterococcus Faecium* | 552,969 | 123,647 |
| *Klebsiella pneumoniae* | 2,199,679 | 116,563 |
| *Acinetobacter baumannii* | 1,100,829 | 101,998 |
| *Pseudomonas aeruginosa* | 902,600 | 234,596 |
| *Enterobacter aerogenes* | 588,854 | 0 |
| *Enterobacter cloacae* | 1,401,037 | 0 |
| *Escherichia coli* | 2,690,162 | 431,252 |


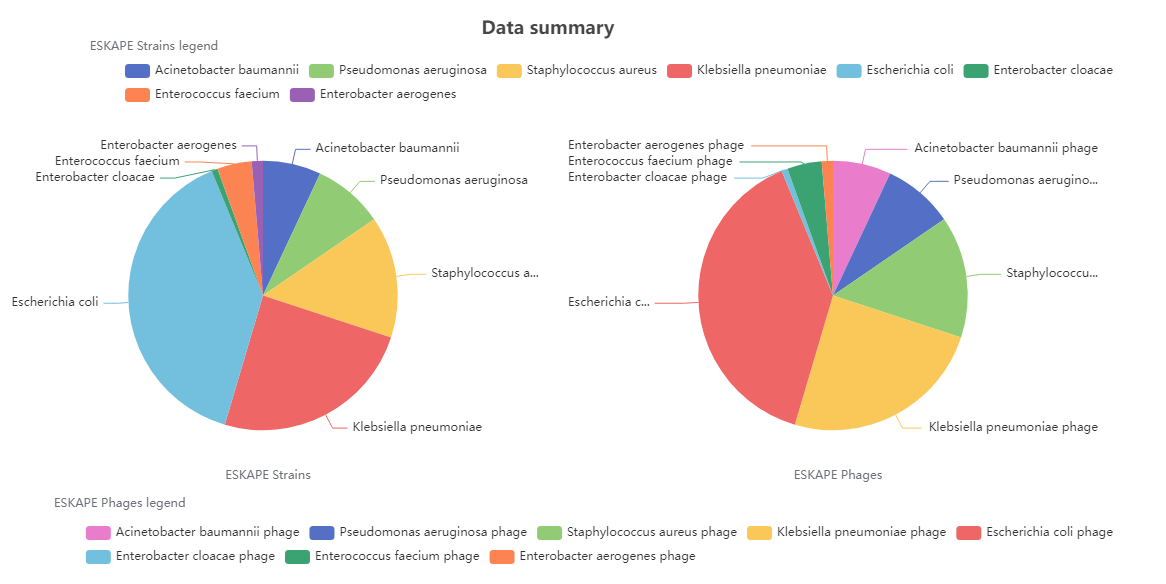
**Figure S1.** **The number and distribution of ESKAPE bacteria and phages.** Each element in the pie chart represents the quantity percentage.


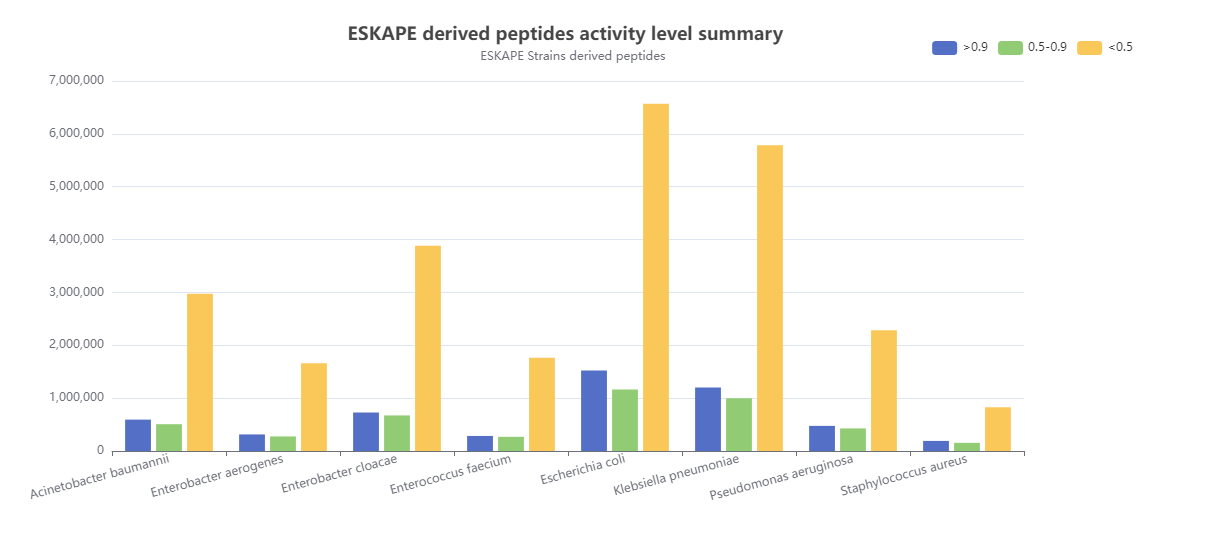
**Figure S2.** **Distribution of different activity levels of AMPs digged from prophages.** In general, activity score less than 0.5 is defined as low activity, activity scores between 0.5 and 0.9 are defined as medium activity, activity scores greater than 0.9 is defined as high activity**.**


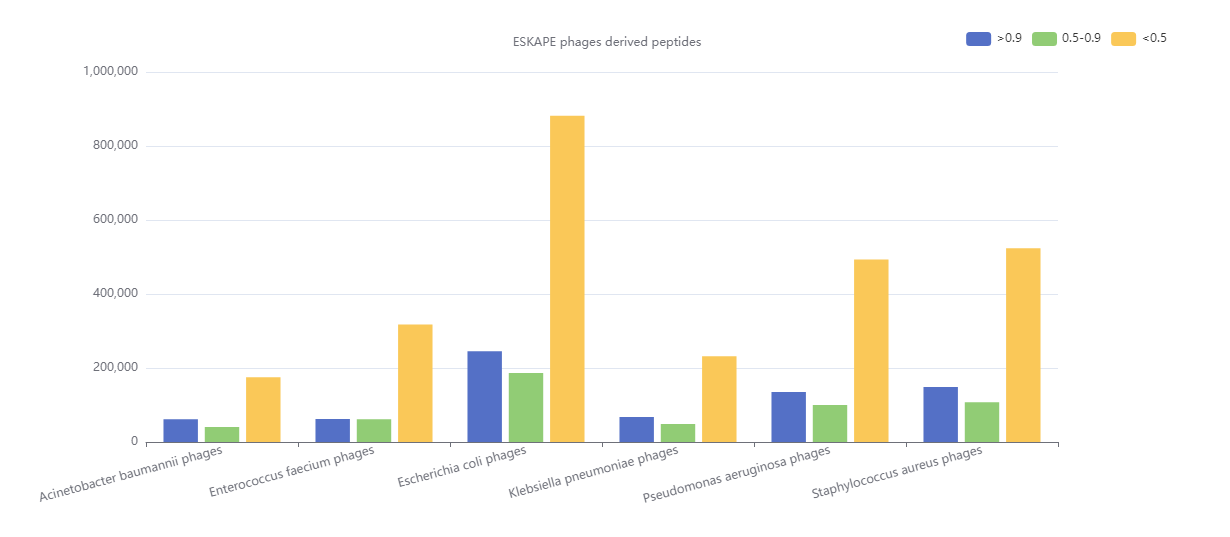
**Figure S3.** **Distribution of different activity levels of AMPs digged from prophages.** In general, activity score less than 0.5 is defined as low activity, activity scores between 0.5 and 0.9 are defined as medium activity, activity scores greater than 0.9 is defined as high activity.

**
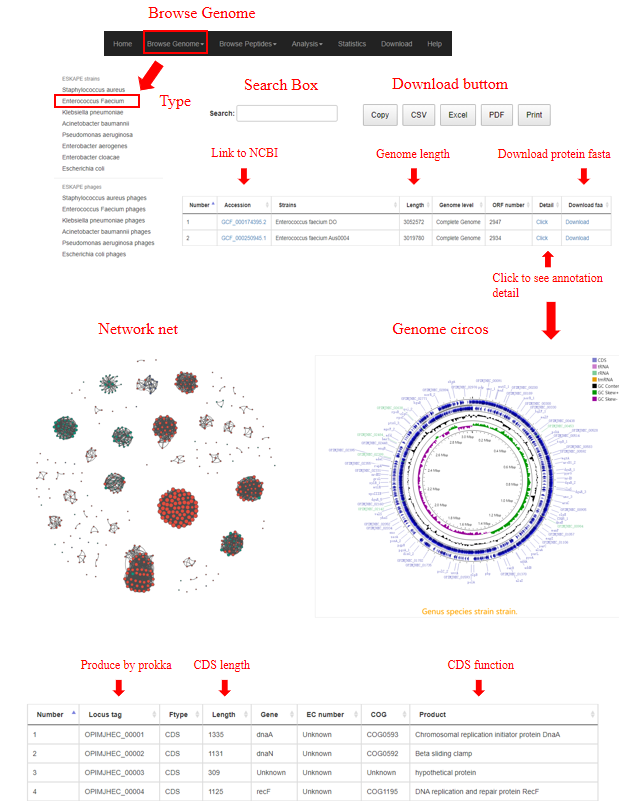
Figure S4.** **The interface of ESKtides.** The ESKtides provided a user-friendly browse mode in which users can browse by strains or phage types, in each table users can search strains accession, genome length, ORF number and download CDS protein fasta. Users can click the drop-down menu or click detail to see annotation detail.


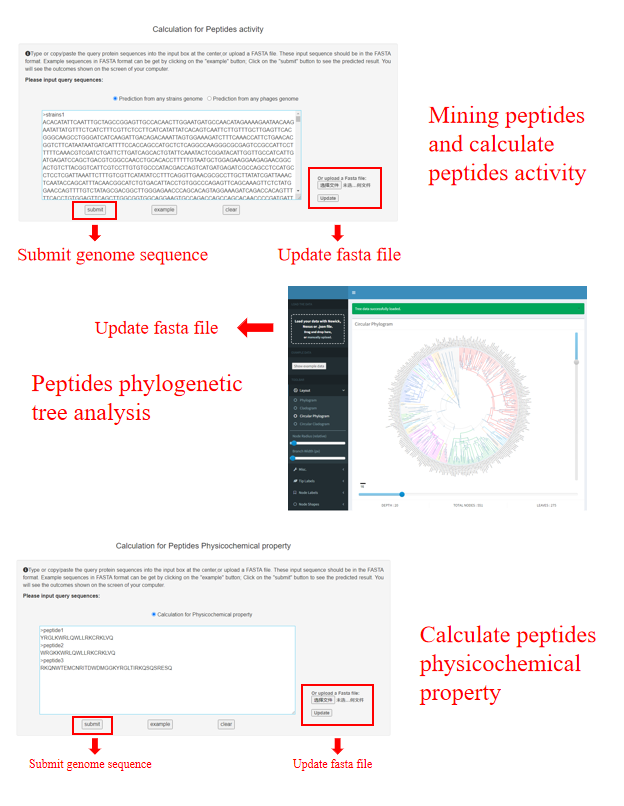
**Figure S5.** **The manual of ESKtides analysis module.** Users can use online analysis modules. Peptides activity Prediction module can dig out peptides and score it by entering the corresponding fasta sequence. Peptides Phylogenetic tree module can provide similarity analysis of peptide sequences and calculating. Physicochemical property module can calculate the physicochemical properties of the peptide sequences.
